# Mapping brucellosis risk in Kenya and its implications for control strategies in sub-Saharan Africa

**DOI:** 10.1038/s41598-023-47628-1

**Published:** 2023-11-18

**Authors:** James M. Akoko, Athman Mwatondo, Mathew Muturi, Lillian Wambua, Hussein M. Abkallo, Richard Nyamota, Caroline Bosire, Stephen Oloo, Konongoi S. Limbaso, Francis Gakuya, Daniel Nthiwa, Andrew Bartlow, Earl Middlebrook, Jeanne Fair, Joseph O. Ogutu, John Gachohi, Kariuki Njenga, Bernard Bett

**Affiliations:** 1 International Livestock Research Institute, Nairobi, Kenya; 2 Zoonotic Disease Unit, Nairobi, Kenya; 3 Department of Medical Microbiology and Immunology, Faculty of Health, University of Nairobi, Nairobi, Kenya; 4 Faculty of Veterinary Medicine, Dahlem Research School of Biomedical Sciences, Freie Universität Berlin, Berlin, Germany; 5 Kenya Medical Research Institute, Nairobi, Kenya; 6 Wildlife Research and Training Institute, Naivasha, Kenya; 7 Department of Biological Sciences, University of Embu, Embu, Kenya; 8 Los Alamos National Laboratory, Los Alamos, NM USA; 9 Biostatistics Unit, Institute of Crop Science, University of Hohenheim, Stuttgart, Germany; 10 Global Health Programme, Washington State University, Nairobi, Kenya; 11 School of Public Health, Jomo Kenyatta University of Agriculture and Technology, Nairobi, Kenya; 12 Paul G, Allen School of Global Health, Washington State University, Pullman, WA 99164 USA; 13 Present Address: World Organisation for Animal Health, Sub-Regional Representation for Eastern Africa, Nairobi, Kenya

**Keywords:** Diseases, Infectious-disease epidemiology

## Abstract

In Sub-Saharan Africa (SSA), effective brucellosis control is limited, in part, by the lack of long-term commitments by governments to control the disease and the absence of reliable national human and livestock population-based data to inform policies. Therefore, we conducted a study to establish the national prevalence and develop a risk map for *Brucella* spp. in cattle to contribute to plans to eliminate the disease in Kenya by the year 2040. We randomly generated 268 geolocations and distributed them across Kenya, proportionate to the area of each of the five agroecological zones and the associated cattle population. Cattle herds closest to each selected geolocation were identified for sampling. Up to 25 cattle were sampled per geolocation and a semi-structured questionnaire was administered to their owners. We tested 6,593 cattle samples for *Brucella* immunoglobulin G (IgG) antibodies using an Enzyme-linked immunosorbent assay (ELISA). We assessed potential risk factors and performed spatial analyses and prevalence mapping using approximate Bayesian inference implemented via the integrated nested Laplace approximation (INLA) method. The national *Brucella* spp. prevalence was 6.8% (95% CI: 6.2–7.4%). Exposure levels varied significantly between agro-ecological zones, with a high of 8.5% in the very arid zone with the lowest agricultural potential relative to a low of 0.0% in the agro-alpine zone with the highest agricultural potential. Additionally, seroprevalence increased with herd size, and the odds of seropositivity were significantly higher for females and adult animals than for males or calves. Similarly, animals with a history of abortion, or with multiple reproductive syndromes had higher seropositivity than those without. At the herd level, the risk of *Brucella* spp. transmission was higher in larger herds, and herds with a history of reproductive problems such as abortion, giving birth to weak calves, or having swollen testes. Geographic localities with high *Brucella* seroprevalence occurred in northern, eastern, and southern regions of Kenya all primarily characterized by semi-arid or arid agro-ecological zones dominated by livestock pastoralism interspersed with vast areas with mixed livestock-wildlife systems. The large spatial extent of our survey provides compelling evidence for the widespread geographical distribution of brucellosis risk across Kenya in a manner easily understandable for policymakers. Our findings can provide a basis for risk-stratified pilot studies aiming to investigate the cost-effectiveness and efficacy of singular and combined preventive intervention strategies that seek to inform Kenya’s Brucellosis Control Policy.

## Introduction

Brucellosis, a zoonotic disease that affects both humans and livestock, poses a significant threat to public health and economic stability in regions where livestock farming is an important source of livelihood^1,2^. While brucellosis is the world's most widespread endemic zoonosis, it also ranks as one of the seven most neglected diseases^3^. The disease also ranks highly in One Health Zoonotic Disease prioritization exercises conducted by African countries, including Kenya, mainly due to its economic impacts, including livestock productivity losses and restrictions on livestock trade^4–6^.

Incidence estimates in humans range between 5 and 12.5 million cases annually, primarily presenting with protracted debility^3^. In Kenya, the government has drawn a Brucellosis National Prevention and Control Strategy, emphasizing the need to develop conceptual frameworks for improving understanding of the spatial distribution and other risk factors to better design and implement programs leading to possible elimination by the year 2040^7^.

Previous brucellosis epidemiological studies have been implemented in small agro-ecological zones, limited administrative units, or community settings. They have also employed different field and diagnostic methodologies^8–11^. These studies show high brucellosis prevalence in pastoral production systems. They also demonstrate that brucellosis exposure decreases as herd size and size of land holding reduce^9^. The high prevalence of brucellosis in pastoral areas has been attributed to occupational and culturally-related risky practices despite local communities having significant knowledge of the disease^9,10, 12^.

Nevertheless, the scope of these research studies is limited geographically and temporally, presenting significant challenges to reliable analyses of animal-level, ecological, meteorological and edaphic correlates of exposure.

To address this knowledge gap, a nationally representative epidemiological study covering multiple regions is required to map brucellosis risk to inform national control strategies and generate hypotheses to guide future research. Previous studies have shown that determining disease burden and developing disease risk maps can enable governments to allocate their limited resources on appropriate prevention and control efforts. For example, using a national Rift Valley fever (RVF) risk map^13^, Kenya implemented enhanced RVF surveillance during the forecasted 2015–2016 RVF high-risk period, focusing on 22 high-risk counties^14^. Here, we designed and implemented a national cattle population-based study using a stratified sampling procedure and testing of 6593 serum samples across five agro-ecological zones and contrasting husbandry systems, covering all cattle age and sex classes to build an evidence base for brucellosis prevention and control in Kenya and possibly elsewhere in sub-Saharan Africa.

## Methods

### Sample size determination

We conducted this national cross-sectional survey in Kenya between November 2020 and August 2021. The study covered all the five agro-ecological zones in Kenya, including agro-alpine, high and medium potential, semi-arid, arid, and very arid zones. Since the study aimed to estimate the *Brucella* seroprevalence at the national level, we estimated the sample size using the standard formula for determining a population proportion^15^. In the absence of reference data, we assumed an a priori prevalence of 50%. Based on this assumption, the initial sample size generated was 384 animals. However, two adjustments were made to ensure robustness of the study population size. The first adjustment accounted for potential clustering of the outcome at the herd level, a characteristic expected of *Brucella* based on previous studies^10,16–18^. We estimated a design effect of 8.2 based on an assumed intra-cluster correlation of 0.3 and a maximum of 25 cattle sampled per herd to meet the expectations of the Central Limit Theorem. This adjustment increased the sample size estimate from 384 to 3,150 animals. The second adjustment aimed to amplify the sample size to account for potential confounding variables. In this step, we assumed that the prediction model for *Brucella* would have at least two continuous predictors, each with a significant correlation of 0.5 with the outcome. Meeting the preceding assumptions required sampling 6,700 animals from 268 herds or sampling points.

### Sampling

**Figure 1:**
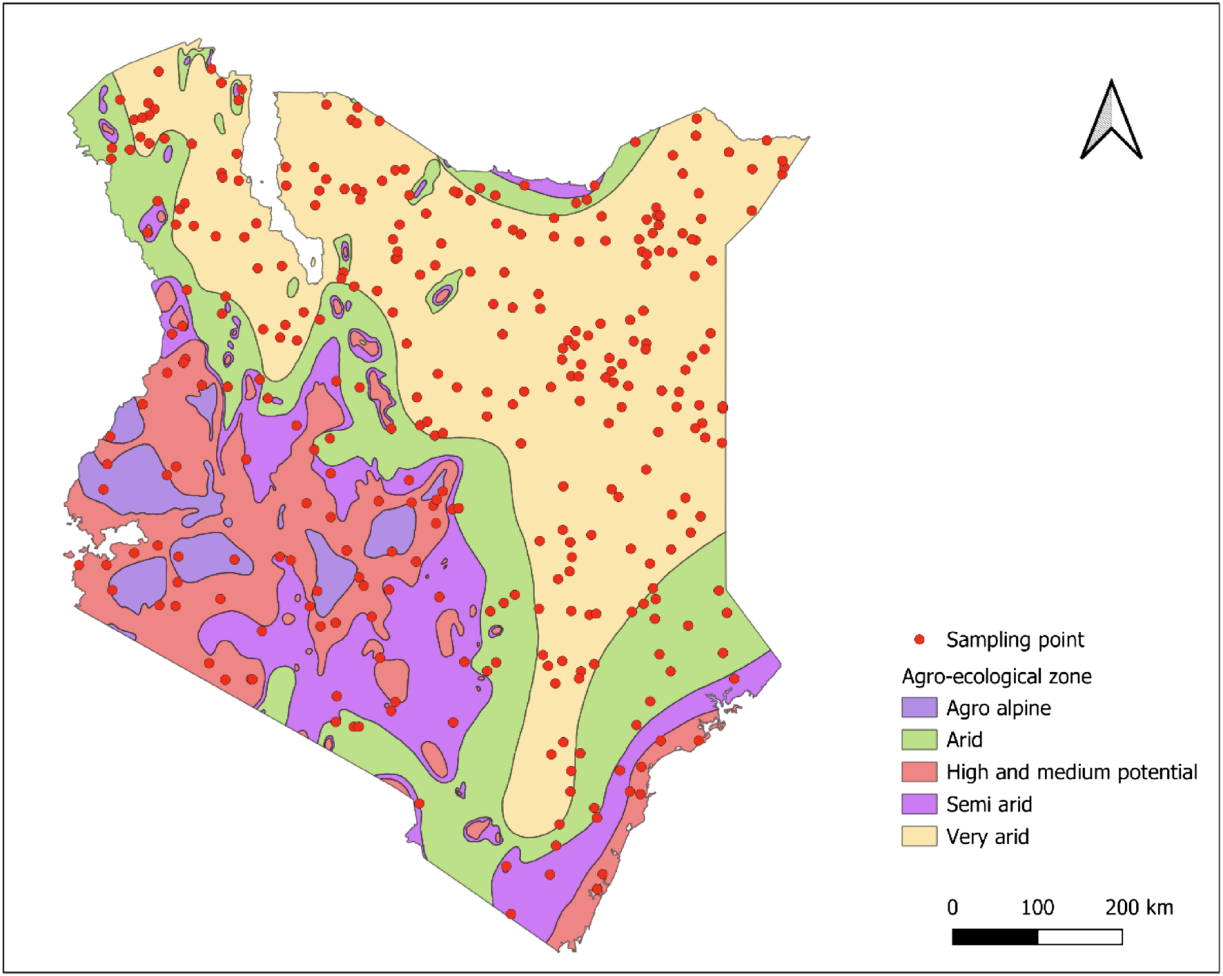
Map of Kenya showing the randomly generated locations for sampling across the different agroecological zones. The map was prepared by Max Korir using QGIS version 3.30.1. The agroecological zone datasets were retrieved from https://geoportal.icpac.net/layers/geonode:ken_aczones (ICPAC Geoportal).

A two-stage sampling technique was employed. The first stage involved selecting households across the country within the five Agro-Ecological Zones (AEZs) using random geographic coordinates (RGC) distributed proportionately to the geographical coverage and livestock population in each AEZ (Fig. 1). In addition, the second stage selected 25 healthy cattle per herd for sampling. After the RGCs were transferred to a handheld GPS to locate their physical locations within Kenya, the closest herds within a 5 km radius of each geolocation were identified for sampling. In the second stage, 25 healthy cattle were selected per herd to be included in the study. For cattle herds with more than 25, the team selected 25 animals randomly. If a chosen herd had fewer than 25 animals, additional nearby herds were included until the desired sample size of 25 animals per geolocation was achieved.

The team then administered a structured questionnaire to the household heads at each sampling point to capture herd-level data, including herd size, herd composition, and the presence or absence of reproductive syndromes within the past two years. For each randomly selected cattle, the questionnaire also captured the demographic characteristics such as age, sex and history of reproduction syndromes before the collection of blood samples.

### Blood sample collection and analysis

Animals were restrained appropriately and about 6 ml of blood was collected from the jugular vein using a barcoded plain vacutainer tube. The blood samples were initially kept for 15 min to allow clotting. They were then transported in a cool box to the field laboratory where serum was extracted after centrifugation at 1300 g for 10 min. The serum samples were aliquoted into barcoded cryovials and kept in a motorized freezer (− 20 ◦C), which was also used to transport the samples to a biosecurity level 2 laboratory at the International Livestock Research Institute (ILRI) in Nairobi. To detect circulating antibodies against *Brucella* spp., samples were analyzed in duplicates using an indirect enzyme-linked immunosorbent assay (ID screen Brucellosis serum Indirect ELISA Multispecies from IDvet innovative diagnostics, France), as per the manufacturer instructions, to detect antibodies against *Brucella abortus*, *Brucella melitensis* and *Brucella suis*. Testing and interpretation of results were done according to the guidelines provided by the manufacturer, with the mean optical density (OD) being used to determine a positive or negative status. The samples were analyzed in a biosecurity level 2 laboratory, as recommended for *Brucella*^19–21^, while following the set biosafety and biosecurity measures for biosafety level 2 laboratory.

### Descriptive and risk factor analysis

We used the R statistical software (version 4.2.3)^22^ for data cleaning and analysis. Descriptive summaries were obtained via cross-classification tables using the *CrossTable* function in *gmodels* package^23^, while risk factor analyses were implemented using Bayesian spatial logistic regression with a logit link and a binomial error distribution fitted using the integrated nested Laplace approximation (INLA) method^24^. The model uses approximate Bayesian inference to estimate the posterior distribution of model parameters. The model was fitted to the data using the R-INLA function in the R-INLA package^25^. INLA was preferred for these analyses because of its computational efficiency and accurate approximation of parameters of a wide class of generalized linear mixed models (GLMM), generalized additive models (GAM), spatial and spatio-temporal models using stochastic partial differential equations (SPDE)^26^. The data were initially analysed using a GLMM, with herd ID as the random effect, but this model was later replaced by a GLMM model with a spatial random effect accounting for spatial autocorrelation.

The spatial logistic regression model is given in Eq. 1. The logit link function ${\eta }_{i}=log\left({\pi }_{i}/\left(1-{\pi }_{i}\right)\right)$ links the probability of animal-level *Brucella* seropositivity ${\pi }_{i}$ to the linear predictor.

$${\eta }_{i}={\beta }_{0}+\sum_{j=1}^{m}{\beta }_{j}{x}_{ji}+f({z}_{i})$$

In the linear predictor, $\beta_{0}$ is the intercept, *m* is the number of covariates, ${\beta }_{j}$ is the regression slope for covariate ${x}_{j}$ and $f(z_{i} )$ is a generic function accounting for spatial random effects. For the GLMM models $f(z_{i} )$ was assumed to be structured according to independent and identically distributed Gaussian random effects (iid) represented by the herd ID. For the spatial model $f(z_{i} )$ was approximated by an SPDE model.

Fitting the spatial Bayesian models and computing their posterior distributions and posterior predictive distributions requires computationally intensive numerical quadrature methods such as Bayesian quadrature or probabilistic numerics. The Integrated Nested Laplace Approximation (INLA) method is particularly well-suited for this purpose because it performs approximate Bayesian inference by directly calculating the posterior densities for Bayesian hierarchical models. A noteworthy and distinct advantage of R-INLA is its high computational efficiency and reliable approximation relative to the widely used Markov Chain Monte Carlo (MCMC) simulation method, even for spatial data with many observations. This enables rapidly fitting and exploring different contending models, gaining deep insights into the data, performing efficient cross-validation and improving transparency through quick code checking and running models by peers^25^. Consequently, several recent studies have applied the SPDE approach implemented in R-INLA, including to neglected tropical diseases, such as visceral leishmaniasis in Ethiopia, onchocerciasis in Africa and Yemen and Ebola in the DRC^26–28^.

### Univariable and multivariable analyses with the GLMM model

The univariable and multivariable models were fitted to the animal- and herd-level data in turns. The predictors considered for the analyses using the animal-level data included an animal’s age, sex and history of abortion, birth of weak calves, or retained placenta. The models that used herd-level data considered the effects of herd size, ecology and occurrence of abortion, birth of weak calves and retained placenta in the index herd. Parameter estimates for variables whose 95% credible intervals excluded zero were considered significant.

We also checked the 95% credible intervals for the estimated variance component to establish if it excluded zero. We built the multivariate model as follows. First, we ranked the univariate models using the Watanabe Information Criterion (WAIC). Second, starting with the best supported univariate model, we added the covariate from the second best supported model and the first-order interaction terms between the variables in the joint model. The second covariate was retained in the model if the WAIC for the joint model was smaller than for the best univariate model. Otherwise, the second covariate was dropeped from the joint model. Next, the covariate from the third best supported model was similarly added to the joint model and the process continued until all the covariates had been considered. Interaction terms higher than the first-order were omitted from the multivatiate model to limit or control the number of estimated parameters relative to the sample size or number of spatial sampling points.

### Spatial analyses

Because it used multiple spatial datasets, we commenced the spatial analysis by developing a causal web diagram to guide the selection of predictors for modelling (Figure S1). Previous studies indicate that *Brucella* is endemic in pastoral and agro-pastoral areas where livestock are raised in large herds, graze communally, and are likely to interact with wildlife^9^. Climatic and environmental factors that influence types of livestock production systems and relevant socioeconomic practices in an area, were therefore considered as antecedent predictors. At the host level, individual characteristics such as species, age, physiological status, and production levels may also influence exposure patterns (Figure S1).

The spatial model was developed through five successive stages. The first involved the development of a mesh over the spatial domain and the second involved the specification of a projector matrix to connect the observed data with the nodes of the mesh (Supplementary text S2). The mesh was constructed using the Kenya shape file to define the location of the domain. We downloaded the shapefile from https://www.diva-gis.org/gdata. The third step involved defining the SPDE model; non-informative priors were specified in this case. The fourth and the fifth stages involved setting up an index for the spatial field and constructing a data stack that was required for fitting the model, respectively.

A total of 49 predictor variables were tested for their association with *Brucella* seropositivity. These included: (a) the host characteristics—sex and age—recorded during sampling; (b) spatial distribution of cattle, camels, sheep and goats based on census data from the Department of Veterinary Services of Kenya, and predictions from the gridded livestock of the world project^27^; (c) environmental variables such as the aridity index, digital elevation indices, slope of the land surface, soil types, and (d) bioclimatic variables. A list of these datasets with a description of their resolutions and sources is provided in Table S3. A variable was considered significant if its 95% credible intervals excluded zero.

### Generating predictions from fitted models

The final model with ecological variables only was used to predict the probability of *Brucella* seroprevalence across the country. A full model with the animal-level factors could not be used for this purpose because there were no data on these characteristics for unsampled locations. We first generated a 5 km grid and centroids from the grid used to extract the predictor variables from relevant raster files to generate this prediction. We then plotted a map of the expected *Brucella* seroprevalence derived from the model predictions. Finally, we extracted and plotted the posterior marginal densities for the range and variance of the random effects. Estimates of these two parameters are relevant for designing future surveys and risk-based surveillance for *Brucella*.

### Ethics approval and consent to participate

The study obtained research approval from the National Commission for Science, Technology, and Innovation (NACOSTI) under reference number NACOSTI REF: 218346. The ILRI's Institutional Animal Care and Use Committee (IACUC) also reviewed our protocols and granted an approval REF: ILRI-IACUC2021-18. The guidelines and regulations established by NACOSTI and IACUC were strictly adhered to during implementation of this study, while only including cattle from herds where informed consent was issued by the household head.

## Results

We sampled 6,593 cattle from 468 herds distributed across the five agro-ecological zones in Kenya. The average number of animals sampled per herd was 14 (range: 1–25). Single herd sampling was achieved for 160 herds, the rest required multiple sampling to attain 25 animals per sampling point. While 44.87% of the sampled herds (210 herds) had more than 20 animals, 50 herds had between 10–19 animals, whereas less than 10 animals were obtained from 208 herds. A total of 449 out of the 6,593 samples tested positive for *Brucella* antibodies, resulting in a national seroprevalence of 6.8% (95% CI: 6.2–7.4%); 31.3% of all the herds had at least one positive animal.

Results from descriptive analyses are presented in Table 1. Adult animals had a higher seroprevalence (10.1%, range 9.1–11.1) than younger animals (3.9%, range 2.9–5.1). Female animals had significantly higher seroprevalence than males. Seroprevalence also differed significantly by herd type. Animals kept in single-species herds had a higher seroprevalence (9.9%, range 8.3–11.5) than animals kept in mixed-species herds (6.1%, range 5.5–6.7). Further, *Brucella* seropositivity was higher in arid AEZ but lower in non-arid AEZ. Animals kept in herds with a history of single or multiple reproductive syndromes also had higher seropositivity. Results of the univariable model run using the INLA package on all the variables are presented in supplimentary material (Table S4).

**Table 1:** Summary of cattle population composition, descriptive characteristics, and *Brucella* spp. seropositivity.

Variable	Category	Total	No. *Brucella spp.* positive	% *Brucella* spp. sero-positive (95% CI)	*p*-value
Sex	Male	1707	70	4.1 (3.2–5.0)	0.001
	Female	4886	377	7.7 (6.9–8.4)	
Age category	Suckling	1138	45	3.9 (2.9–5.1)	0.001
	Weaner	1059	31	2.9 (2.1–3.9)	
	Waiting to breed	984	27	2.7 (1.8–3.7)	
	Adult	3068	344	10.1 (9.1–11.1)	
Herd type	Cattle only	130	31	32.8 (16.9–31.1)	0.050
	Cattle mixed with others	338	112	33.1 (28.1–38.3)	
Herd size	1–25	252	16	6.3 (3.9–9.4)	< 0.001
	26–100	114	60	52.6 (43.8–62.4)	
	More than 100	102	67	26.4 (20.0–34.3)	
History of reproduction problems within a herd	No	296	48	16.2 (12.5–20.6)	< 0.001
	Yes	172	95	55.2 (48.3–63.4)	
History of abortion within a herd	No	414	118	28.5 (24.2–32.9)	0.012
	Yes	54	25	53.7 (40.7–66.9)	
History of weak calf within a herd	No	457	135	29.5 (25.4–33.8)	0.006
	Yes	11	8	72.3 (54.5–100.0)	
History of swollen testis in a herd	No	462	139	30.1 (25.9–34.4)	0.137
	Yes	6	4	66.7 (50.0–100.0)	
Retained placenta	No	448	140	31.3 (27.0–35.7)	0.195
	Yes	20	3	15.0 (5.0–31.5)	
5 agro-ecological zones	Agro Alpine	52	0	0.0 (0.0–3.1)	< 0.001
	High and medium potential	151	8	5.2 (2.6–8.9)	
	Semi-arid	40	15	37.5 (25.0–54.0)	
	Arid	61	27	44.3 (32.8–57.6)	
	Very arid	164	93	56.7 (49.4–64.8)	
Categorized AEZ	Non-Arid	203	8	3.9.4 (2.0–6.7)	< 0.001
	Arid	265	135	50.9 (44.9–57.3)	

NB: The *p*-value presented in this trable are generated from a chi-square test.

The results of the ILNA model used to analyze the animal-level data are summarized in Table 2. They show the risk of *Brucella* spp. seropositivity to be higher for female than male animals. Also, the adult animals had an elevated risk of being exposed to *Brucella* than the suckling calves and other age categories.

**Table 2:** Significant risk factors associated with *Brucella* positivity at the individual animal level, identified by the INLA model with household as the random effect.

Variable	Category	Mean	SD	Percentile range	
				2.5%	97.25%
Animal sex	Female	1.0 (Ref.)			
	Male	− 0.281	0.161	− 0.601	0.031
Age category	Adults	1.0 (Ref.)			
	weaners	− 1.340	0.213	− 1.772	− 0.935
	Waiting to breed	− 1.314	0.219	− 1.760	− 0.899
	Suckling calves	− 0.875	0.187	− 1.250	− 0.518

The estimated associations between the known syndromes of brucellosis and the frequency of detection of *Brucella* antibodies in female adult cattle are summarized in Table 3. Female adult animals with a history of abortion, and those that had experienced more than one syndrome of brucellosis had a higher likelihood of being positive for *Brucella* antibodies.

**Table 3:** Summary of syndromes associated with brucellosis in female adult cattle based on results of the nonspatial INLA model with household as the random effect.

Variable	Category	Positivity for *Brucella* antibodies		Mean and standard deviation (SD)		Percentile range	
Syndromes of brucellosis in livestock		n/N	% seropositivity and (95%CI)	Mean	SD	2.5%	97.5%
Retained placenta	No	317/2978	10.6 (9.6–11.7)	1 .0 (Ref.)			
	Yes	11/79	13.9 (7.6–21.6)	0.362	0.372	− 0.401	1.059
History of abortion	No	291/2917	10.0 (8.9–11.1)	1. 0 (Ref.)			
	Yes	29/140	26.4 (20.0–34.3)	0.852	0.251	0.352	1.336
History of weak calf	No	323/3028	10.7 (9.6–11.8)	1. 0 (Ref.)			
	Yes	5/29	17.2 (6.9–31.3)	0.771	0.582	− 0.428	1.858
Multiple syndromes	No	316/3009	10.5 (9.4–11.5)	1.0 (Ref.)			
	Yes	12/48	25.0 (14.6–37.7)	1.106	0.421	0.257	1.910

The herd-level risk factors associated with *Brucella* spp. seropositivity are summarized in Table 4. Herds containing animals with a history of reproductive syndromes such as abortions, retained placenta, or the birth of weak calves were significantly associated with *Brucella* spp. seropositivity, relative to those kept in herds with no history of reproductive syndromes. Herds kept in non-arid areas were less likely to get exposed to *Brucella* spp. infection as opposed to those reared in arid and semi-arid AEZs. The risk of exposure to *Brucella* spp. was also significantly lower for small (≤ 25), than for middling (26–99) or large (≥ 100) herds.

**Table 4:** Summary of results of the INLA model for herd level factors.

Variable	Category	Mean	SD	Percentile range	
				2.5%	97.5%
Herd size	26–99 animals	1.0 (Ref.)			
	1–25 animals	1.758	0.355	1.752	2.471
	100 and above	2.162	0.369	2.156	2,901
History of any reproduction problem (abortion, retained placenta or weak calf)	No	1.0 (Ref.)			
	Yes	1.151	0.266	0.633	1.675
Ecological zone	Arid- and semi-arid	1.0 (Ref.)			
	Non-arid areas	− 0.2.044	0.423	− 2.918	− 1.256

### Brucellosis risk mapping

**Figure 2:**
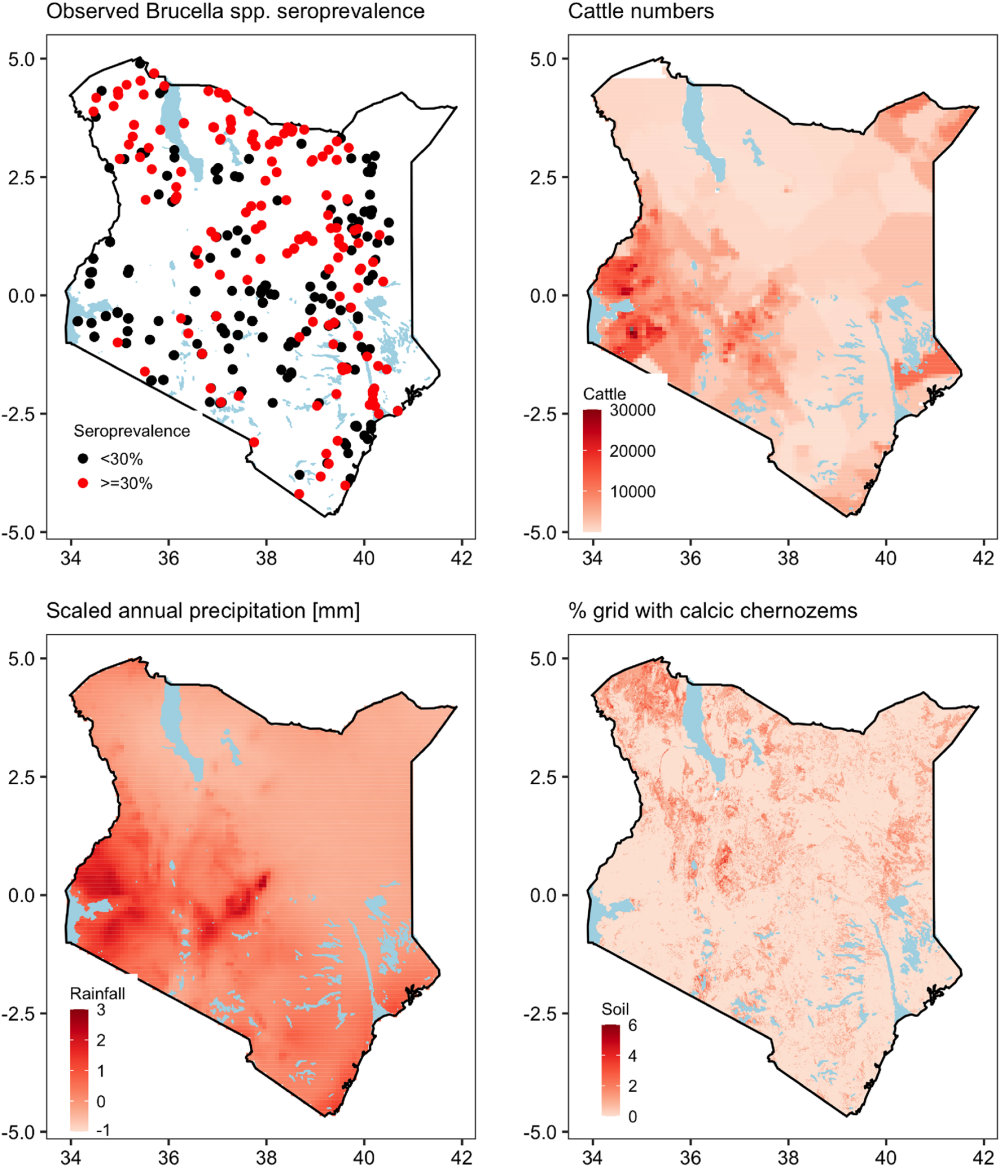
Maps of the observed distribution of *Brucella* spp. seropositivity in cattle (**a**), and of the variables that were significant in the final model (cattle numbers (**b**), annual precipitation (**c**) and calcic chernozems (**d**)) fitted to the *Brucella* data.

From the 6,593 records collected from 468 herds in this study, only 6,587 (99.9%) records with complete entries were used for spatial analysis. Relatively high *Brucella* spp. seroprevalence was observed in the northern parts of Kenya (Fig. 2a). In contrast, low seroprevalence was observed in the central highlands and the western parts of the country (Fig. 2a). Areas with the highest seroprevalence had relatively lower cattle densities (Fig. 2b), lower rainfall (Fig. 2c) and calcic chernozems soil types (Fig. 2d).

### Univariable modelling

Of the 53 variables used in the univariable analysis, 36 showed significant associations with seroprevalence (Table S5). Including the spatial random effects in this analysis reduced the number of significant variables to 19.

### Multivariable analysis

Table S6 summarises the outputs from the final multivariable model with animal and environmental variables, while Table S7 provides results from the model with only the environmental variables as significant predictors. In both cases, the spatial effect improved the model fit. This was manifested by substantial reductions in the Deviance Information Criterion (DIC) from 2,992.5 to 2,600.7 and 3125.8 to 2701.8 in the first and second models, respectively.

The DIC, used to compare the relative fit of the different Bayesian hierarchical models further revealed that including animal-level factors improved the model fit. The age of an animal was significantly associated with *Brucella* spp. seropositivity as the level of exposure increased with age. Regarding environmental factors, areas with lower rainfall and those with lower cattle numbers were associated with higher log odds of *Brucella* spp. seropositivity than those with high rainfall and cattle numbers (Table S6, Table S7). All the continuous variables satisfied the linearity assumption. All the first-order interaction terms were insignificant.

### Model predictions

**Figure 3:**
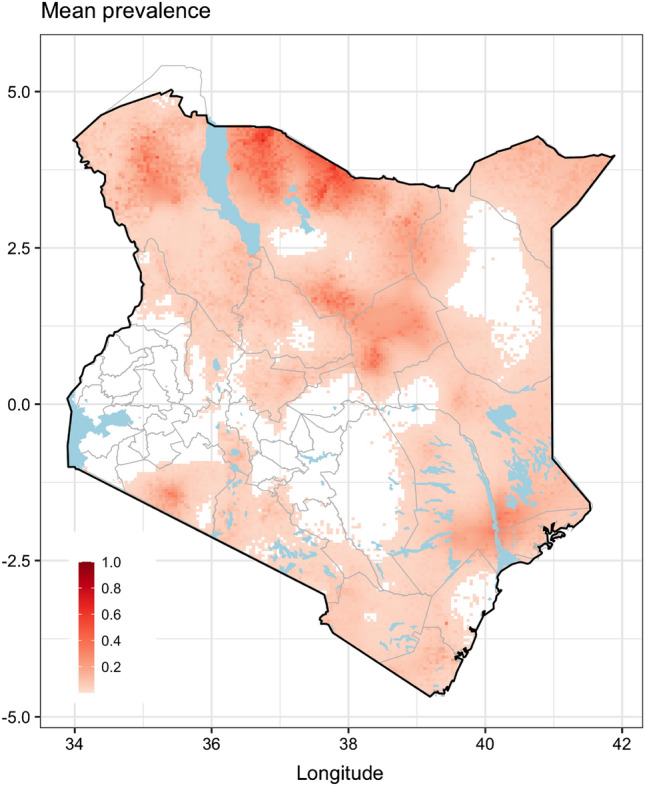
Predicted expected *Brucella* spp. seroprevalence in Kenya.

Figure 3 illustrates the predicted expected *Brucella* spp. seroprevalence in Kenya with high predicted seroprevalence occurring in the northern, eastern, and southern regions, all primarily characterized by semi-arid, arid or very arid agro-ecologies dominated by livestock pastoralism and mixed livestock-wildlife systems. Conversely, we predicted very low expected seroprevalence for the central and the western highlands and the Lake Victoria Basin region, all characterized by humid and high and medium potential agro-ecologies dominated by varying levels of intensified and mixed crop-livestock production systems.

## Discussion

To our knowledge, this is the first national brucellosis exposure assessment for Kenya. Designed to be representative at the national level, we coupled risk factor assessment with risk mapping, thus gaining a comprehensive epidemiological picture and expanding our understanding of animal brucellosis exposure patterns in the diverse Kenyan territory. We found a consistent pattern of higher exposure among adult animals, females, and those raised primarily under pastoralism. In addition, our risk mapping confirmed that the disease risk is concentrated mainly in the hard-to-reach Arid and Semi-Arid Lands (ASALs), occupying three-quarters of the country's land surface and home to about 36%, 70% and 90% of human, livestock and wildlife populations, respectively. Although developing countries have made remarkable progress in brucellosis control, vast endemic areas such as Kenya's limit proper surveillance and control systems, calling for concerted efforts to launch "One Health"-led control programs tailored to realities of particular geographical and socio-cultural contexts.

The high seroprevalence found in the drier agroecological zones is consistent with findings of studies conducted in smaller geographical locations^9,10,28,29^. In these zones, pastoralist communities keep livestock communally in large mobile herds, constantly searching for pasture and water, thereby sharing common environments. Unfortunately, this management practice brings infected aborting and birthing animals into close contact with susceptible livestock, promoting the transmission of *Brucella*. Since pastoralism is a way of life for these communities, studies are beginning to characterize livestock movement networks that could inform disease prevention and control practices and policies^30^. Indeed, previous studies have established strong correlations between human brucellosis in these regions and *Brucella* seroprevalence in livestock from engaging in risky practices that promote *Brucella* infection^9^. Specifically, pastoralists consume raw milk from infected animals that constantly shed bacteria in milk and also assist infected animals during parturition without protective clothing or equipment^12^.

The low brucellosis seroprevalence in the high agricultural potential agro-alpine zones and high and medium potential zones was considerably lower than the seroprevalence in the drier ASAL zones relative to the national prevalence of 6.8%. This low seroprevalence is linked to the husbandry systems that focus on intensifying milk production and targeting high-value markets in nearby urbanizing regions^31^. Intensification is a response to increasing human population density and land scarcity, pushing livestock farmers to keep few high-value livestock breeds under the stall-feeding system^32^, limiting between-herd contacts, and thus tremendously decreasing the transmission risk of *Brucella* via placental or fetal tissues and urine. Furthermore, the production system in these zones employs biosecurity measures that cull animals with poor reproductive performance and minimize the introduction of animals from outside the farm through within-herd replacement of breeding animals^31^. This also curtails the within-herd spread of *Brucella* to humans.

The risk map we generated here can inform research into the effectiveness and efficacy of singular or combined preventive interventions, shifting away from costly one-size-fits-all control strategies that demotivate policymakers towards more focused management based on risk stratification. Thus, the map is a holistic instrument for multifaceted strategies that may, for instance, inform pilot vaccination studies and community-based trialing of public health education and promotion.

The risk of *Brucella* spp. exposure was higher in females than males and in adult than juvenile cattle. These findings are consistent with those of previous studies and are primarily attributed to longer exposure time among females due to their role in providing replacement animals and milk production^10^. Additionally, in pastoralist communities, bull calves are often disposed of at an early age, leaving few males for breeding, and herds typically consist of less than 30% males^33^.

While Kenya is implementing the national Brucellosis Control Strategy, the strategy's background and rationale should be motivated by the livestock-dependent economy in the > 75% of the country's landmass besides reducing the threat to human health. Although this study excluded the more populous brucellosis-susceptible small stock, our data show the distribution patterns of *Brucella* spp. exposure and indicate that the probable economic impact of animal brucellosis in the country is sufficient to motivate policymakers to increase funding for its control measures. Moreover, previous studies have estimated positive economic benefits from and cost-effectiveness of such control measures undertaken in brucellosis-endemic settings elsewhere^34^. Not surprisingly, many countries have drawn up brucellosis eradication programs, with some employing vaccination-only policies, others using test-and-slaughter-only policies and some using both approaches and complementing them with other measures^3^. This likely reflects the occupation and culture prevalent in particular localities, with little consideration given to the role of human-livestock-environment interface interdependencies that facilitate disease transmission. It follows that that village-level community health volunteer and community animal health worker teams, attentive to particular social, cultural, livestock husbandry and disease control contexts, should form the cornerstone for the integrating socio-cultural environment into One Health practice in those remote, hard-to-reach zones. Anthropology can provide important additional context to One Health practice, which often overlooks the perspectives and lived experiences of communities affected by zoonoses spillovers to humans^35^. Such a strategy would contribute to achieving Sustainable Development Goal #3 of the United Nations, which focuses on equity and commitment to attending to peoples’ health in hard-to-reach areas^36^.

Policies, experts, and modelling exercises suggest that vaccination efforts alone are insufficient or take nearly three decades to control brucellosis effectively31. Even so, the B. abortus S19 vaccine is recommended as a live attenuated vaccine for female calves aged between 3 and 6 months, the age group highly treasured by pastoralists. It is administered as a single subcutaneous dose or as a reduced dose of organisms to adult cattle. This vaccine effectively reduces the incidence of brucellosis in cattle herds31. Thus, governments would do well to pilot it in pastoralist herds that value female cattle over males in SSA countries that are yet to adopt vaccination as a comprehensive brucellosis control strategy, particularly in areas with high-risk populations.In conclusion, this is the first national population-based survey of Brucellosis seroprevalence for a livestock species in SSA. The findings are valuable to the Kenya Brucellosis national prevention and control strategy that has set elimination targets by 2040. The use of seroprevalence mapping offers a methodologically rigorous approach for targeting surveillance, prevention, control, piloting disease control interventions and furthering research.

### Supplementary Information

Supplementary Figure S1.

Supplementary Information 2.

Supplementary Table S3.

Supplementary Table S4.

Supplementary Table S5.

Supplementary Table S6.

Supplementary Table S7.

## Data Availability

All the data are included in this article and its supplementary files.

## References

[1] Pappas G, Papadimitriou P, Akritidis N, Christou L, Tsianos EV (2006). The new global map of human brucellosis. Lancet Infect. Dis..

[2] Addis M (2015). Public health and economic importance of brucellosis: A review. Public Policy Adm. Res..

[3] Franc KA, Krecek RC, Häsler BN, Arenas-Gamboa AM (2018). Brucellosis remains a neglected disease in the developing world: A call for interdisciplinary action. BMC Public Health.

[4] Munyua P (2016). Prioritization of zoonotic diseases in Kenya, 2015. PLoS ONE.

[5] Sekamatte M (2018). Multisectoral prioritization of zoonotic diseases in Uganda, 2017: A one health perspective. PLoS ONE.

[6] Pieracci EG (2016). Prioritizing zoonotic diseases in Ethiopia using a one health approach. One Heal..

[7] Republic of Kenya Zoonotic Disease Unit (2014). National Strategic Plan for the Implementation of One Health Kenya.

[8] Akoko J (2020). Serological and molecular evidence of Brucella species in the rapidly growing pig sector in Kenya. BMC Vet. Res..

[9] Osoro EM (2015). Strong association between human and animal brucella seropositivity in a linked study in Kenya, 2012–2013. Am. J. Trop. Med. Hyg..

[10] Kairu-Wanyoike S (2019). Positive association between Brucella spp. Seroprevalences in livestock and humans from a cross-sectional study in Garissa and Tana River Counties, Kenya. PLoS Negl. Trop. Dis..

[11] Kadohira M, McDermott JJ, Shoukri MM, Kyule MN (1997). Variations in the prevalence of antibody to brucella infection in cattle by farm, area and district in Kenya. Epidemiol. Infect..

[12] Njenga, M. K. *et al.* Comparison of knowledge, attitude , and practices of animal and human brucellosis between nomadic pastoralists and non- pastoralists in Kenya. *BMC Public Health* 1–10 (2020). 10.1186/s12889-020-8362-0 PMC7041083 32093689

[13] Munyua, P. M. *et al.* Predictive factors and risk mapping for rift valley fever epidemics in Kenya. *PLoS ONE* **11**, (2016). 10.1371/journal.pone.0144570 PMC4726791 26808021

[14] Oyas H (2018). Enhanced surveillance for Rift Valley Fever in livestock during El Niño rains and threat of RVF outbreak, Kenya, 2015–2016. PLoS Negl. Trop. Dis..

[15] Dohoo IR, Martin SW, S. H. *Methods in Epidemiologic Research. Charlottetown, Prince Edward Island, Canada, VER Inc. 2012.* (2012).

[16] Njeru J, Nthiwa D, Akoko J, Oyas H, Bett B (2021). Incidence of Brucella infection in various livestock species raised under the pastoral production system in Isiolo County. Kenya. BMC Vet. Res..

[17] Akoko JM (2021). Molecular epidemiology of Brucella species in mixed livestock-human ecosystems in Kenya. Sci. Rep..

[18] Otte MJ, Gumm ID (1997). Intra-cluster correlation coefficients of 20 infections calculated from the results of cluster-sample surveys. Prev. Vet. Med..

[19] Reddy, M. Laboratory Biosafety and Biosecurity for Handling Transboundary Animal Disease and Zoonotic Emerging Pathogens (2019).

[20] OIE. Brucellosis (B. abortus, B.melitensis and B. suis). (2016).

[21] Hussain T (2017). Seroprevalence of brucellosis in ovines of Ganderbal district of Kashmir Valley. J. Entomol. Zool. Stud..

[22] R Core Team. An Introduction to dplR. *Ind. Commer. Train.* **10**, 11–18 (2008).

[23] Steven-, A. M. *et al.* Package ‘ epiR ’. (2023).

[24] Lindgren F, Rue H (2015). Journal of statistical software bayesian spatial modelling with R-INLA. J. Stat. Softw..

[25] Blangiardo M, Cameletti M, Baio G, Rue H (2013). Spatial and spatio-temporal models with R-INLA. Spat. Spatiotemporal. Epidemiol..

[26] Lindgren, F., Rue, H. & Lindström, J. An explicit link between gaussian fields and gaussian markov random fields: The stochastic partial differential equation approach. *J. R. Stat. Soc. Ser. B Stat. Methodol.* **73**, 423–498 (2011).

[27] Robinson, T. P. *et al.* Mapping the global distribution of livestock. *PLoS ONE* **9**, (2014). 10.1371/journal.pone.0096084 PMC4038494 24875496

[28] Lokamar PN (2022). Prevalence of brucellosis in livestock keepers and domestic ruminants in Baringo County, Kenya. PLOS Glob. Public Health.

[29] Muema, J. *et al.* Sero_-_epidemiology_of_brucellosis_in_people_and_t.

[30] VanderWaal, K., Gilbertson, M., Okanga, S., Allan, B. F. & Craft, M. E. Seasonality and pathogen transmission in pastoral cattle contact networks. *R. Soc. Open Sci.* **4**, (2017). 10.1098/rsos.170808 PMC5749993 29308225

[31] Perry BD, Grace D, Sones K (2013). Current drivers and future directions of global livestock disease dynamics. Proc. Natl. Acad. Sci. USA.

[32] Herrero M (2013). The roles of livestock in developing countries. Animal.

[33] Gachohi, J. M., Njenga, M. K., Kitala, P. & Bett, B. Modelling vaccination strategies against rift valley fever in livestock in Kenya. *PLoS Negl. Trop. Dis.* **10**, (2016). 10.1371/journal.pntd.0005049 PMC5156372 27973528

[34] Kiiza D (2023). A systematic review of economic assessments for brucellosis control interventions in livestock populations. Prev. Vet. Med..

[35] Steffens TS, Finnis E (2022). Context matters: Leveraging anthropology within one health. One Health.

[36] Bangert M, Molyneux DH, Lindsay SW, Fitzpatrick C, Engels D (2017). The cross-cutting contribution of the end of neglected tropical diseases to the sustainable development goals. Infect. Dis. Poverty.

