# Supplementary material for: Mapping brucellosis risk in Kenya and its implications for control strategies in sub-Saharan Africa
Source: Sci Rep. 2023 Nov 18;13:20192. doi: 10.1038/s41598-023-47628-1 (PMC10657468; doi:10.1038/s41598-023-47628-1)
Supplement: Supplementary file 1 — Supplementary Figure S1. [file 41598_2023_47628_MOESM1_ESM.pdf]

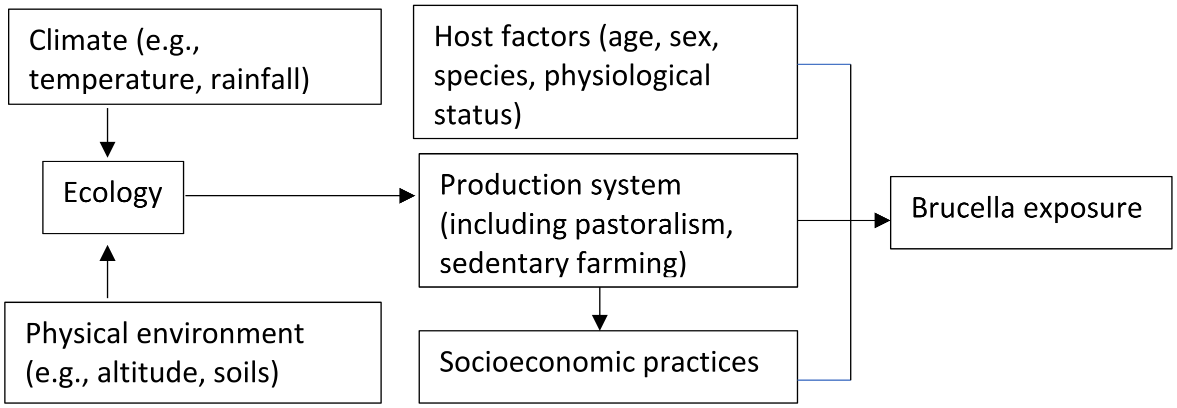

Figure S1. Causal web diagram illustrating relationships between the factors that were considered for *Brucella* spp. statistical modelling
