# Supplementary material for: Mapping brucellosis risk in Kenya and its implications for control strategies in sub-Saharan Africa
Source: Sci Rep. 2023 Nov 18;13:20192. doi: 10.1038/s41598-023-47628-1 (PMC10657468; doi:10.1038/s41598-023-47628-1)
Supplement: Supplementary file 2 — Supplementary Information 2. [file 41598_2023_47628_MOESM2_ESM.pdf]

Spatial analysis _Supplementary Text S2

The spatial effect was estimated using stochastic partial differential equations (SPDE). The SPDE model assumes that the outcome of interest has a multivariate Gaussian distribution with a mean of zero and a variance-covariance structure that can be specified using Matérn variance function. The R-INLA package that was used in this work provides a practical approach for implementing this analysis. It approximates Gaussian Random Field (GF) to Gaussian Markov Random Field (GMRF) through five successive stages (Lindgren and Rue 2015) as follows: (i) discretization of the spatial domain using a Delaunay triangulation to generate a mesh over the spatial domain, (ii) the specification of a projector matrix to connect the observed data with the mesh created (space mapping), (iii) the specification of the SPDE model, (iv) setting up of a data stack and (v) the specification of the INLA model.

The mesh was constructed with the Kenya shape file being used to set location of the domain. The shapefile was downloaded from https://www.diva-gis.org/gdata. The key parameters that were specified were the lengths of the edges of the triangles in the inner and outer extension of the spatial domain (beyond the boundary of the spatial domain) of 0.5- and 5-degree units. At the same time, a cut off value of 0.5-degree units was supplied to define the minimum distance that could be allowed between any two points. Points that were closer to each other than allowed by this parameter were replaced by a single vertex in the mesh. The mesh therefore provided a neighbourhood structure with a dimension, m of 946 x 946 nodes.

Given that the SPDE model approximates the GF at the vertices of the mesh, a projector matrix should be created to map the response onto the mesh. This was accomplished by generating the projector matrix that links the locations of the responses, using the spatial coordinates, with the vertices of the mesh. The dimension of the matrix was 6,587 (the number of records used in the analysis) by 946 (the number of vertices of the mesh). The SPDE model was set up using the function *inla.spde2.matern*() to provide a solution for the continuous domain spatial Markov random fields described by Lindgren and Rue (Lindgren and Rue 2015). The last two procedures for setting up the model involve establishing a data stack and specifying the code for the *inla*() function.

Running the INLA models

Based on the illustration given in Figure 1, a total of 49 predictor variables were tested for their association with *Brucella* seropositivity. These included: (a) the host characteristics -- sex and age – that were recorded during sampling; (b) spatial distribution of cattle, camels, sheep and goats based on census data from the Department of Veterinary Services and predictions from the grided livestock of the world project (Robinson *et al.* 2014); (c) environmental variables such as the aridity index, digital elevation indices, slop of the land surface, soil types, and (d) bioclimatic variables. A list of these datasets with a description of their resolutions and sources is provided in Supplementary material 1.

Univariable analyses were performed (with each predictor being used in turns) to identify significant variables that could be selected for multivariable analyses. Variables whose 2.5 - 97.5% credible intervals excluded zero were considered as being significant. The

univariable analyses were implemented without the spatial random effects. Subsequently, univariable models that generated significant results were ran again with the random effects.

Multivariable analyses included variables that were significant through the two steps of analysis described above. A combination of backwards and forwards variable selection procedure involving all the variables that were significant at univariable stage of the analysis was used to develop a multivariable model. The significance of the first order interaction terms between variables that were significant in the final model were determined. Models with and without the animal level factors were developed given that the latter was required for predicting Brucella seroprevalence across the spatial domain. The significance of the spatial random effect (the SPDE model) was evaluated using deviance information criterion (DIC) statistic.

Generating predictions from fitted models

The final model that had ecological variables only was used to predict Brucella seroprevalence across the country. A full model with the animal-level factors could not be used for this purpose because there were no data on these characteristics in unsampled locations. To generate this prediction, a 5km grid was first generated and centroids from the grid used to extract the predictor variables from relevant raster files. The mean Brucella seroprevalence from the predictions made by the model were then plotted out to obtain a seroprevalence map.

Finally, the marginals densities for range and variance were extracted from the model results and plotted. The results illustrated by these two parameters are relevant for designing future surveys and risk-based surveillance for Brucella.
