# Supplementary material for: Mapping brucellosis risk in Kenya and its implications for control strategies in sub-Saharan Africa
Source: Sci Rep. 2023 Nov 18;13:20192. doi: 10.1038/s41598-023-47628-1 (PMC10657468; doi:10.1038/s41598-023-47628-1)
Supplement: Supplementary file 3 — Supplementary Table S3. [file 41598_2023_47628_MOESM3_ESM.pdf]

## S3 Table. GIS data and their sources

Candidate predictor variables descriptions

| **Variable (source)** | ***Units*** |
|---|---|
| Slope (derived from GOTOPO30) | degrees |
| 1km resolution Mean temp warmest quarter (University of York, AfriClim) | $^oC*10$ |
| 1km resolution Mean temp coolest quarter (University of York, AfriClim) | $^oC*10$ |
| 1km resolution Mean annual rainfall (University of York, AfriClim) | mm |
| 1km resolution Rainfall wettest month (University of York, AfriClim) | mm |
| 1km resolution Precipitation of Driest Month (University of York, AfriClim) | mm |
| 1km resolution Rainfall seasonality (University of York, AfriClim) | mm |
| 1km resolution Rainfall wettest quarter (University of York, AfriClim) | mm |
| 1km resolution Rainfall driest quarter (University of York, AfriClim) | mm |
| 1km resolution Mean diurnal range in temp (University of York, AfriClim) | $^oC*10$ |
| 1km resolution Isothermality (University of York, AfriClim) | $^oC*10$ |
| 1km resolution Temperature Seasonality (University of York, AfriClim) | $^oC*10$ |
| 1km resolution Max temp warmest month (University of York, AfriClim) | $^oC*10$ |
| 1km resolution Min temp coolest month (University of York, AfriClim) | $^oC*10$ |
| 1km resolution Annual temperature range (University of York, AfriClim) | $^oC*10$ |
| 250m resolution Soil organic carbon density (depth 0 cm) (ISRIC) | kg/m3 |
| 5 arc-minute Gridded Livestock density (Harvard Dataverse) | animals per km2 |
| 250 m resolution Soil texture fraction at depth 0.00 m | factor |
| 30-arc seconds digital elevation model (USGS) | meters |
| 250 m resolution Silt content (2-50 micrometer) at depth 0.00 m (ISRIC) | mass fraction % |
| 250m resolution sand content (50-2000 micrometer) depth 0.00m | mass fraction (%) |
| 250 m resolution Soil pH x 10 in H2O at depth 0.00 m (ISRIC) | Index*10 |
| 250 m resolution Calcic vertisols WRB class (ISRIC) | % |
| 250 m resolution Calcic gypsisols WRB class (ISRIC) | % |
| 250 m resolution Calcic gleysols WRB class (ISRIC) | % |
| 250 m resolution Calcic histisols WRB class (ISRIC) | % |
| 250 m resolution Calcic kastanozems WRB class (ISRIC) | % |
| 250 m resolution Calcic luvisols WRB class (ISRIC) | % |
| 250 m resolution Calcic regosols WRB class (ISRIC) | % |
| 250 m resolution Calcic solonetz WRB class (ISRIC) | % |
| 250 m resolution Calcic chernozems WRB class (ISRIC) | % |
| 250 m resolution Haplic chernozems WRB class (ISRIC) | % |
| 250 m resolution Haplic solonetz (ISRIC) WRB class (ISRIC) | % |
| 250 m resolution gleyic solonetz (ISRIC) WRB class (ISRIC) | % |
| 250 m resolution gleyic solonchaks (ISRIC) WRB class (ISRIC) | % |
| 250 m resolution luvic chernozems (ISRIC) WRB class (ISRIC) | % |
| 250 m resolution mollic solonetz (ISRIC) WRB class (ISRIC) | % |
| 250 m resolution petric calcisols (ISRIC) WRB class (ISRIC) | % |

| | |
|---|---|
| Land cover 2015 | |
| Mammal diversity | |

Data sources and reference

| *Data* | *Source* | *Reference* |
|---|---|---|
| *Bioclimatic (temperature; precipitation; seasonal variables)* | **University of York, AfriClim:** https://webfiles.york.ac.uk/KITE/AfriClim/GeoTIFF_30s/baseline_worldclim/ | [6] |
| *Soil type, Soil properties* | **ISRIC:** https://www.isric.org/explore/soilgrids | [5] |
| *Elevation [GOTOPO30)* | **USGS:** https://earthexplorer.usgs.gov/ | [7] |
| *Cattle density* | **HAVARD, Dataverse:** https://dataverse.harvard.edu/dataset.xhtml?persistentId=doi:10.7910/DVN/GIVQ75 | [4] |
| *Slope* | Derived from Elevation (GOTOPO30) | |

REFERENCES


1. Gilbert M, Nicolas G, Cinardi G, Van Boeckel TP, Vanwambeke SO, Wint GW, et al. Global cattle distribution in 2010. V3 ed: Harvard Dataverse; 2018.
2. Hengl T, de Jesus JM, Heuvelink GB, Gonzalez MR, Kilibarda M, Blagotić A, et al. SoilGrids250m: Global gridded soil information based on machine learning. PLoS One. 2017;12(2):e0169748.
3. New M, Lister D, Hulme M, Makin I. A high-resolution data set of surface climate over global land areas. In: Climatic Research Unit UoEA, editor. A high-resolution data set of surface climate over global land areas: Climatic Research Unit; 2002.
4. Platts PJ, Omeny PA, Marchant R. AFRICLIM: high-resolution climate projections for ecological applications in Africa. AFRICLIM: high-resolution climate projections for ecological applications in AfricaThe University of York; 2015.
5. USGS. Global 30 Arc-Second Elevation (GTOPO30). In: USGS, editor. 1996.
