# Supplementary material for: Mapping brucellosis risk in Kenya and its implications for control strategies in sub-Saharan Africa
Source: Sci Rep. 2023 Nov 18;13:20192. doi: 10.1038/s41598-023-47628-1 (PMC10657468; doi:10.1038/s41598-023-47628-1)
Supplement: Supplementary file 4 — Supplementary Table S4. [file 41598_2023_47628_MOESM4_ESM.pdf]

Table S4: Univariable analysis of risk factors associated with *Brucella* positivity as analyzed by ILNA model, with household as a random effect for animal level factors (sex, and age category), and ward for herd level factors.

Variable	Category	Mean	Standard deviation	Percentile range	
				**2.5%**	**97.25**
**Sex**	Female	Ref			
	Male	-0.819	0.144	-1.108	-0.542
**Age category**	Adult	Ref			
	weaner	-0.453	0.204	-1.868	-1.067
	Waiting to breed	-0.386	0.216	-1.826	-0.979
	Suckling	-0.990	0.176	-1.343	-0.654
**Herd type**	Cattle only	Ref			
	Cattle mixed with others	0.097	0.420	-0.733	0.920
**Herd size**	26-100	Ref			
	1-25	-1.700	0.314	-2.340	-1.109
	More than 100	0.554	0.233	0.099	1.015
**History of reproduction problems within a herd**	No	Ref			
	Yes	1.715	0.321	1.094	2.355
**History of abortion within a herd**	No	Ref			
	Yes	0.990	0.473	0.075	1.931
**History of weak calf within a herd**	No	Ref			
	Yes	0.783	0.878	-0.879	2.570
**History of swollen testis in a herd**	No	Ref			
	Yes	0.599	0.718	-0.827	1.996
**Multiple syndromes**	No	Ref			
	Yes	1.606	0.373	0.882	2.347
**History of retained placenta in a herd**	No	Ref			
	Yes	0.000	0.31.623	-62.017	62.017

**5 agro-ecological zones**	Agro alpine	Ref			
	High and medium potential	4.926	5.692	-3.555	18.211
	Semi-arid	7.641	5.704	-0.880	20.943
	Arid	7.946	5.702	-0.576	21.242
	Very arid	8.307	5.691	-0.186	21.583
**Zone**	Arid	Ref			
	Non-arid	-3.614	0.495	-4.662	-2.721
