# Supplementary material for: Mapping brucellosis risk in Kenya and its implications for control strategies in sub-Saharan Africa
Source: Sci Rep. 2023 Nov 18;13:20192. doi: 10.1038/s41598-023-47628-1 (PMC10657468; doi:10.1038/s41598-023-47628-1)
Supplement: Supplementary file 5 — Supplementary Table S5. [file 41598_2023_47628_MOESM5_ESM.pdf]

Table S5: Results of spatial analysis done on variables using univariable model

Category	Variable	Levels	Significant without spatial random effect	Significant with spatial random effect
Host characteristics	Sex	Female, Male	Y	Y
	Age	Calf, Weaner, Yearling, Adult	Y	Y
	Altitude		Y	N
	Slope		N	N
	Land cover	LC high, moderate, low	Y	N
Domography	Human population		N	Y
	Sheep numbers		Y	N
	Goats numbers		Y	Y
	Camel numbers		Y	Y
	Indigenous cattle numbers		Y	Y
	Exotic cattle numbers		Y	N
	Gridded cattle population		Y	N
	Gridded goat population		Y	N
	Mammalian diversity		N	N
Soils	Calcic gleysols		N	N
	Calcic gypsisols		N	N
	Calcic histosols		N	N
	Calcic kastanozems		Y	N
	Calcic luvisols		Y	N
	Calcic regosols		Y	N
	Calcic solonetz		N	Y
	Calcic vertisols		Y	N
	Calcic chernozems		Y	Y
	Gleyic solonetz		N	N
	Haplic calcisols sodic		N	Y
	Haplic calcisols		Y	N
	haplic chernozems		Y	N
	Haplic gypsisols		N	N
	Haplic solonetz		Y	N
	Luvic calcisols		N	N
	Luvic chernozems		Y	N
	Mollic solonetz		N	N
	Putric calcisols		Y	N
Climate	Aridity		Y	Y
	Annual mean temperature		Y	Y
	Mean diurnal range		N	N
	Isothermality		N	N
	Temperature seasonality		N	N
	Maximum temperature - warmest month		Y	Y
	Minimum temperature - coldest month		Y	N

Temperature annual range	N	N
Mean temperature -- wettest quarter	Y	Y
Mean temperature -- driest quarter	Y	N
Mean temperature -- warmest quarter	Y	Y
Mean temperature -- coldest quarter	Y	Y
Annual precipitation	Y	Y
Precipitation of the wetest month	Y	Y
Precipitation of the driest month	Y	N
Precipitation seasonality	N	N
Precipitation of the wetest quarter	Y	Y
Precipitation of the driest quarter	Y	N
Precipitation of the warmest quarter	Y	N
Precipitation of the coldest quarter	Y	Y
