# Supplementary material for: Mapping brucellosis risk in Kenya and its implications for control strategies in sub-Saharan Africa
Source: Sci Rep. 2023 Nov 18;13:20192. doi: 10.1038/s41598-023-47628-1 (PMC10657468; doi:10.1038/s41598-023-47628-1)
Supplement: Supplementary file 6 — Supplementary Table S6. [file 41598_2023_47628_MOESM6_ESM.pdf]

Table S6. Posterior distribution of ecological parameters that were found significant in the hierarchical Bayesian model that was fitted to national Brucella seropositivity data from Kenya

Variable	Mean	SD	2.5% quantile	97.5% quantile
Intercept	-2.84	0.38	-3.58	-2.09
Annual precipitation	-1.23	0.57	-2.44	-0.18
Calcic chernozems	0.29	0.15	0.00	0.59
Cattle numbers	-0.02	0.01	-0.04	0.00
