# Supplementary material for: Mapping brucellosis risk in Kenya and its implications for control strategies in sub-Saharan Africa
Source: Sci Rep. 2023 Nov 18;13:20192. doi: 10.1038/s41598-023-47628-1 (PMC10657468; doi:10.1038/s41598-023-47628-1)
Supplement: Supplementary file 7 — Supplementary Table S7. [file 41598_2023_47628_MOESM7_ESM.pdf]

Table S7. Posterior distribution of the parameters for the hierarchical Bayesian model that was fitted to national Brucella seropositivity data from Kenya

Variable	Mean	SD	2.5% quantile	97.5% quantile
Intercept	-2.43	0.37	-3.16	-1.70
Female	-0.23	0.16	-0.55	0.08
Male	0.00			
Calf	-0.86	0.19	-1.23	-0.50
Yearling	-1.27	0.22	-1.72	-0.86
Weaner	-1.27	0.21	-1.70	-0.87
Adult	0.00			
Annual precipitation	-1.31	0.56	-2.50	-0.28
Calcic chernozems	0.31	0.15	0.01	0.61
Cattle numbers	-0.02	0.01	-0.03	0.00
